# Optimizing Mechanical and Microstructural Properties of Sandy Clayey Silt Stabilized with Lignin Fiber and Cement Synergy

**DOI:** 10.3390/polym17111584

**Published:** 2025-06-05

**Authors:** Shuangfeng Guo, Xiaoyi Jiang, Zhihua Zhang, Qingrui Lu, Zhe Wang, Kai Zhao

**Affiliations:** 1 College of Transportation Engineering, Nanjing Tech University, Nanjing 211816, China; shuangfengguo@njtech.edu.cn (S.G.); zhaokai@njtech.edu.cn (K.Z.); 2 Chongqing University Industrial Technology Research Institute, Chongqing 400039, China; 3 School of Civil &Architecture Engineering, East China University of Technology, Nanchang 330013, China; yt_jiangxiaoyi@126.com; 4 Changjiang River Scientific Research Institute of Changjiang Water Resources Commission, Wuhan 430010, China; 5 Beijing Municipal Road & Bridge Building Material Group Co., Ltd., Beijing 100176, China; wzzwangzhe@163.com

**Keywords:** engineering waste soil, sandy clayey silt, lignin fiber, cement, unconfined compressive strength, microstructure

## Abstract

Soil treatment with natural materials is an effective method to improve the mechanical properties of the original soil for recycling engineering construction. This research aims to evaluate the synergistic effects of lignin fiber and cement on sandy clayey silt stabilization. A factorial experimental design was employed, testing five lignin fiber contents (0%, 2%, 4%, 6%, and 8%) and three cement contents (0%, 2%, and 4%) across four curing periods (1, 7, 14, and 30 days). Unconfined compressive strength (UCS) tests were conducted in triplicate for each combination (total *n* = 180 samples), and failure surfaces were analyzed using Scanning Electron Microscopy with Energy Dispersive X-ray spectroscopy (SEM-EDX). Results indicate a critical lignin fiber threshold of 4%, beyond which UCS decreased by 15–20% due to increased void ratios. Statistical analysis (ANOVA, *p* < 0.05) confirmed significant interactions between lignin fiber, cement content, and curing time. For instance, 4% lignin fiber and 4% cement yielded a 139% UCS increase after 30-day curing compared to untreated soil. SEM-EDX revealed that lignin fiber networks enhance ductility by bridging soil particles, while cement hydration reduced particle detachment. These findings provide a quantitative framework for optimizing lignin fiber-cement stabilization in sustainable geotechnical applications.

## 1. Introduction

Silty clay, as an engineering waste soil in subway construction, has been reused in engineering backfills, such as foundation ditches for house construction and subgrades. Before reuse, the silty clay should be treated to improve its stabilization by adding some additives such as crushed stone, tailing sand, fly ash, fiber, etc., due to its low strength and high collapsibility [1,2,3]. Some previous researchers used chemical methods to reinforce it, including the use of cement, lime, curing agents, glass, microorganisms, etc. [4,5]. However, due to the high proportion of chemical amendments, the soil is far beyond the scope of its purification [1,2].

Cement is widely used to improve the strength of silty clay. The homogenous admixture of soil and cement can be conveniently pumped into construction sites from transport vehicles. After adding cement, the products of the hydration reaction can form the cement skeleton in the soil matrix, which can significantly improve the properties of silty clay (i.e., stiffness, compressive strength, etc.) [6,7]. Another aspect is that the high stiffness of cemented soil is brittle and has a low tensile strength as the cement content increases [5,8,9]. Many researchers have reported that the addition of fiber to soil or cemented soil not only improves its compressive strength and tensile strength, but also changes the frictional resistance and occlusion force between fiber and soil particles, which leads to the strong ductility of the soil [10,11,12].

Currently, several kinds of fiber, including natural fibers such as lignin [13,14,15,16], coir [17], banana [18], corn husk [19,20], jute [12,21], wheat straw [22], rice straw [23], cellulose [24,25,26], etc., and artificially synthetic fibers, such as PET [27], polypropylene [28,29], lignosulfonate [15], nylon [30], polyester [31], glass [32], etc., have been used to reinforce engineering waste soil. Artificial synthetic fibers are undesirable because of their expensive cost and environmental pollution; however, natural fibers have the advantages of being inexpensive, abundant, eco-friendly, and biodegradable, and are usually used [33].

Lignin fiber is widely used because its material properties can improve the crack resistance, durability, and toughness of asphalt pavement, concrete, and soil reinforcement. Fan et al. [13] explored the strength characteristics and micro mechanisms of expansive soil treated with different mass fractions of lignin fibers before and after freeze-thaw cycles and found that the tensile capacity of lignin fiber could limit the deformation of the surrounding soils, thus effectively improving the cohesion of the expansive soil. Chen et al. [14] found that the fiber formed a three-dimensional grid structure in the soil samples, which reduced the impact of freeze-thaw on the pore damage, thus enhancing the strength of the soils. Vakili et al. [15] studied the effect of lignin fiber on seasonally frozen expansive soil by conducting cyclic triaxial tests. The results showed that the addition of lignin fiber improves the frost and thaw resistance of expansive soil. Lignin fiber has a high specific surface area and it can easily form “bridges” or spatial network structures surrounding the soil particles to improve soil stabilization [13,14,15,34]. Therefore, the microstructure of fiber-cement stabilized soil requires more attention [35,36]. The selection of lignin fiber and cement as stabilizing agents stems from their complementary roles: cement hydrates to form a cohesive matrix, while lignin fibers bridge microcracks and enhance ductility [37,38]. This synergy addresses the inherent brittleness of cemented soils and the strength reduction observed in fiber-reinforced systems, offering a balanced solution for sustainable infrastructure. Recent advancements in polymer-concrete composites have demonstrated significant progress in enhancing mechanical performance and sustainability. Lee et al. [39] developed a lignin-epoxy hybrid polymer composite that showcases lignin’s potential as a multifunctional additive in construction materials. Similarly, Zhang et al. [40] utilized recycled polyethylene terephthalate (PET) fibers modified with nano-silica to reinforce clayey soils, and Smith et al. [41] highlighted polylactic acid (PLA)-lignin blends for soil stabilization.

Over the last few decades, Scanning Electron Microscopy (SEM), Magnetic Resonance Imaging (MRI), CT scanning, and X-ray diffraction (XRD) have gained popularity in revealing the microstructure of fiber-cement-stabilized soil [20,42]. Vakili et al. [15] used SEM and XRD to elucidate the volumetric variation of expansive soil stabilized by coal ash and cement and found that the formation of cementitious products and ettringite crystals plays a significant role in improving compressive strength. Duong et al. [20] evaluated the microstructure of polypropylene fiber-lime soil during the compressive and tensile tests. The results showed that lots of calcium silicate is attached to the surface of the polypropylene fibers, which leads to the improvement of the cementing force between the internal soil particles and fibers.

Although the microstructure of fiber-cement-stabilized soil, especially using lignin fiber and SEM technology, has been explored in many areas, few studies have investigated the behavior of lignin fiber-cement-stabilized silty clay in regard to life cycle strength, pore structure, and soil particle variation. According to previous studies, lignin fiber could improve the ductility and toughness of silty clay; however, the strength of treated silty clay decreased as the content of lignin fiber increased [14]. Cement could undoubtedly improve the strength of silty clay; however, it changed the behavior of treated silty clay from ductile to brittle. Therefore, we hypothesize that there is an improved balance state of treated sandy clayey silt used in our study (the soil on the border between silt and clay based on [43]), with threshold contents of lignin fiber and cement causing the sandy clayey silt to exhibit relatively large strength and high ductility. There are two critical gaps that need to be investigated: (1) the synergistic mechanisms of lignin fiber and cement remain poorly understood, particularly for transitional soils like sandy clayey silt, and (2) existing studies lack quantitative thresholds for balancing strength and ductility. To verify this hypothesis, in the present study, the effects of lignin fiber content, cement content, and curing time on the physical and mechanical properties of improved soil are studied through an unconfined compressive strength test. The impact mechanism of various admixture materials on the strength and deformation characteristics of treated sandy clayey silt soil is analyzed through SEM. This study addresses these gaps by systematically evaluating the interplay between lignin fiber (0–8%), cement (0–4%), and curing time, revealing a critical fiber content (4%) that optimizes performance.

## 2. Materials and Methods

### 2.1. Experimental Materials

(1) Sandy clayey silt

Sandy clayey silt was taken from a cross-river tunnel along the Yangtze River in Hangzhou City, Zhejiang Province, China (geographic coordinates: 30°15′12″ N, 120°12′36″ E). Samples were retrieved from 2.5–4.0 m below the ground surface within the excavation trench. The procedure for determining natural density is to apply Vaseline to the inside of the ring cutter evenly, remove the soil sample by pressing the blade of the ring cutter vertically downwards, level the upper and lower surfaces of the ring cutter, and weigh the mass of the soil and ring cutter. The process of calculating the natural moisture content was to weigh the wet soil and the aluminum box, then weigh the dry soil and the aluminum box after 8 h of drying in the oven (the temperature is 20 ± 2 °C), and to use the moisture content formula to determine the natural moisture content. In this test, the specific gravity of sandy clayey silt was determined by the specific gravity bottle method by taking 15 g of dry soil and placing it in a 100 mL short-necked specific gravity bottle and measuring the specific gravity of the soil particles with pure water. The liquid limit and plastic limit were determined by using a combined liquid-plastic limit tester. The basic properties of sandy clayey silt are shown in Table 1.

**Figure 1:**
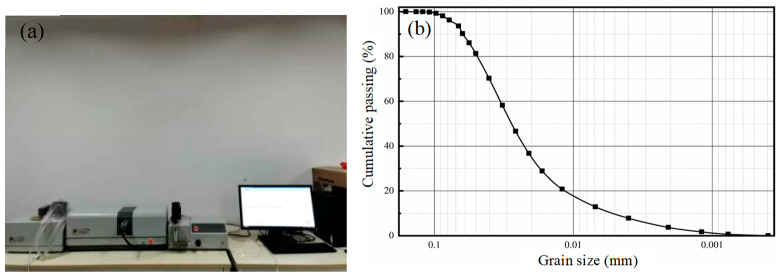
(**a**) Testing Laser particle size distributor; (**b**) Particle size distributions of sandy clayey silt.

In this paper, a BT-9300LD Laser Particle Sizer Analyzer (LPSA) meter was used to measure the particle size distribution of powdery clay, as shown in Figure 1a,b. The testing steps are:

(1) Dry the air-dried and crushed powdered clay through a sieve.
(2) Turn on the computer and laser particle size analyzer and run the program.
(3) After the instrument absorbs water and removes air bubbles, use a small spoon to add the appropriate amount of dispersal sodium phosphate to a small amount of the sample.
(4) Record the test data and analyze the test results. The grain gradation of sandy clayey silt is shown in Table 2, and its particle size distribution is shown in Figure 1b. In Table 2, d_90_, d_60_, d_30_, and d_10_ are particle diameters of the soil mass, which are less than the particle sizes of 0.09 mm, 0.06 mm, 0.03 mm, and 0.01 mm accounting for the total soil mass, respectively. Cu and Cc are the nonuniform coefficient and curvature coefficient, respectively.

(2) Treated sandy clayey silt

Lignin fiber has advantages such as high durability, poor hydrophilicity, a large contact area with soil, improved crack resistance and heat preservation in concrete, and easy degradability. Cement, a calcined product of limestone, clay, and iron ore powder, enhances the strength of sandy clayey silt through hydration reactions and the formation of cementitious bonds. Therefore, different amounts of lignin fiber and cement with different contents are utilized and mixed into the sandy clayey silt.

**Figure 2:**
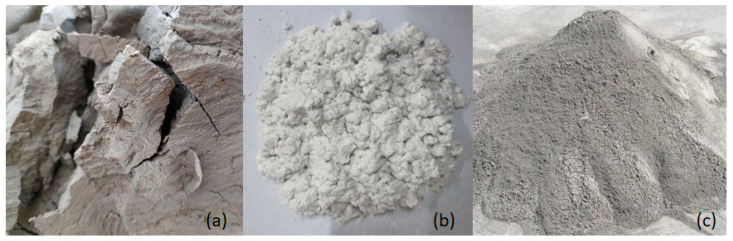
(**a**) Original sandy clayey silt; (**b**) lignin fiber; (**c**) cement.

The cement type is ordinary Portland cement C32.5. The physical properties of lignin fiber were length of 6–8 mm, density of 0.8 g/cm^3^, ash content of 18 ± 5%, pH of 6.5–8.5, and heat resistance of 230 °C. The sandy clayey silt, lignin fiber, and cement are shown in Figure 2.

The mechanical behaviors and deformation responses of blended sandy clayey silt are governed by the synergistic interaction between the chemical hydration processes of cement and the physical interlocking mechanisms of lignin fibers, with their respective material properties (e.g., tensile strength, stiffness, hydration kinetics) and dosages (0–8% lignin fiber, 0–4% cement) acting as key determinants of the composite’s macrostructural performance. Therefore, to evaluate the influence of different contents of lignin fiber and cement on sandy clayey silt, five different contents of lignin fiber (0%, 2%, 4%, 6%, and 8%), three different contents of cement (0%, 2%, and 4%), and four different curing periods (1 d, 7 d, 14 d, and 30 d) were designed. The cement and lignin fiber contents were calculated as percentages of the dry soil mass, and all specimens were cured under controlled conditions (20 ± 2 °C, 95 ± 3% humidity). For more readability in the following text, the mixed sandy clayey silt with different contents of lignin fiber and cement and different curing periods are named in Table 3. For example, the symbol M4S4D30 shows that the contents of lignin fiber M and cement S are 4% and 4%, respectively, and the curing period D is 30 d.

### 2.2. Experimental Measurements and Curing

(1) Unconfined Compressive Test

The unconfined compressive strength (UCS) test can be used to analyze the ultimate strength of soil to resist axial pressure, which is of great significance to the analysis of strength and deformation of improved soil in practical applications. Therefore, in this paper, the UCS test is conducted to analyze the influence of different mixed contents of lignin fiber and cement on mixed sandy clayey silt in strength and deformation characteristics.

**Figure 3:**
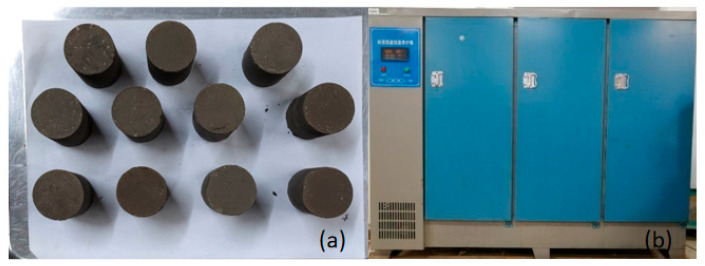
(**a**) Prepared soil samples; (**b**) standard curing box.

UCS tests are carried out in accordance with the ASTM D2166/D2166M-16 (2016) [44]. The test processes, including sample mixing with different materials, sample preparation, and UCS testing, are as follows:

(1) The masses of lignin fiber, cement, sandy clayey silt, and water are weighed according to the ratio designed in the test scheme.
(2) Mix the lignin fiber, cement, and sandy clayey silt in dry condition manually for 1 min to make each material contact evenly. Then, add an appropriate amount of water to the dried-mixed material, and use an electric mixer to mix for 3–5 min with a moisture content of 24%.
(3) Each sample was compacted in three equal layers, with each layer subjected to 25 blows using a standardized rammer (2.5 kg mass, 300 mm drop height). The final density of all samples was maintained within ±1.5% of the target value (1.66 g/cm^3^).
(4) Prepare the samples with a size of 39.1 × 80 mm (diameter × height) by using a triaxial sampler. Then put the samples into the standard constant temperature and humidity curing box for the specified curing period. The prepared soil samples and standard curing box are shown in Figure 3.
(5) When the sample reaches the curing period (1 d, 7 d, 14 d, or 30 d), the YYW-2 strain-controlled unconfined compressor is utilized with an axial strain rate of 1%/min. The finally loaded axial strain is controlled at 3–5% when the axial stress reaches the peak or the stable state. During loading, the axial stress and axial strain are measured and recorded.
(6) Samples cured for 30 d are selected for SEM-EDX analysis to evaluate the stabilized microstructure at hydration equilibrium, ensuring representative insights into long-term performance. This aligns with ASTM C192 guidelines and prior studies on cementitious stabilization. It is conducted in Section 3.

(2) SEM-EDX analysis

**Figure 4:**
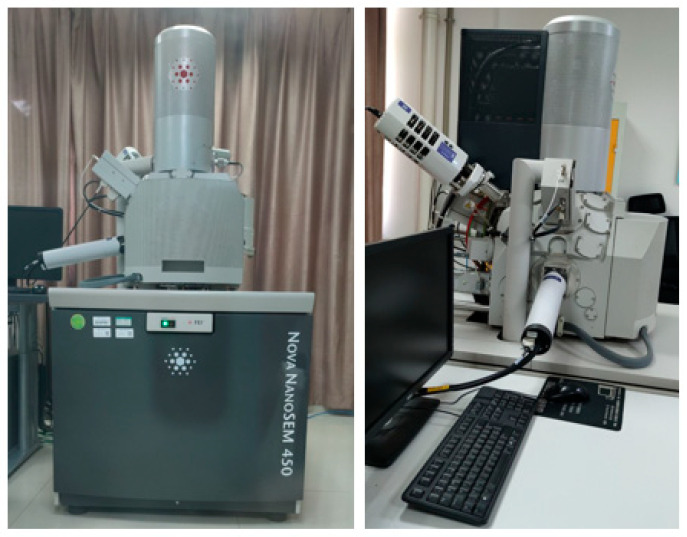
Scanning Electron Microscope (SEM) model FEI Nova NanoSEM 450.

Scanning Electron Microscopy equipped with an Energy Dispersive X-ray analyzer (FEI Nova NanoSEM-EDX 450, FEI Company, Hillsboro, OR, USA) is widely used to analyze the microstructures of physical material, such as the distribution, size, and shapes of soil particles, void size, failure form, and cementation state. SEM is utilized in the present study to observe the microstructure changes of the treated sandy clayey silt as a result of different lignin fiber and cement contents. EDX can be used for qualitative and quantitative analysis, i.e., to identify the type of elements in a part of the sample and the percentage of each element, enabling qualitative and even quantitative elemental analyses of the sample surface. When Scanning Electron Microscopy (SEM) is used in conjunction with an Energy Dispersive X-ray analyzer (EDX), X-rays can also be used as a method to obtain the crystalline information of the samples under consideration. The type of SEM-EDX is FEI Nova NanoSEM-EDX 450 (FEI Company, Hillsboro, OR, USA), as shown in Figure 4, which includes an energy dispersed X-ray analyzer (Inca Energy X-Max20, Oxford Instruments, Abingdon, Oxfordshire, UK), automatic ion sputtering coater (QUORUM Q150R S, Quorum Technologies Ltd., Lewes, East Sussex, UK), and type II cathode-luminescent tube (Gatan ChromaCL, Gatan, Inc., Pleasanton, CA, USA). Samples were gold-coated to enhance conductivity, and EDX mapping identified elemental compositions (e.g., Ca, Si) to verify cement hydration. This approach directly links microstructural features (e.g., fiber networks, ettringite crystals) to macroscopic strength and ductility trends.

Three replicate samples per condition were subjected to SEM-EDX analysis post-failure. Imaging regions were selected based on low-magnification surveys (100×) to identify characteristic failure zones, followed by high-magnification (3000×) examination of three randomly chosen ROIs per sample. Edge regions (≤2 mm) were excluded to avoid preparation artifacts. Instrument parameters (15 kV, 10 mm WD) and calibration protocols were standardized to ensure consistency.

To observe the microstructure of the fracture surface directly, a chosen piece of each sample after compressive failure after 30-d curing in unconfined compressive tests are selected. The SEM-EDX sample should be treated by dressing the fracture surface, drying, and vacuum metalizing. First, the dried samples are fixed on the loading platform and then placed in the gold spraying instrument for vacuum metalizing. This is because the surface of the sample needs to be conducive for scanning by the SEM-EDX instrument, and the fully dried samples cannot be conductive. Then, put the sample (after vacuum metalizing) into the SEM-EDX instrument and take the observation images under 3000 magnifications for subsequent analysis.

### 2.3. UCS Tests and Data Analysis

During the UCS tests, the axial strain and axial stress are recorded to evaluate the improvement in the characteristics of treated sandy clayey silt under the combination of lignin fiber and cement with different contents. The axial strain ε is determined as the ratio of axial deformation and the initial height of the sample with the following equation:

(1) $$\varepsilon =\frac{∆h}{h_{0}}\times 100\%$$

where ∆h is the axial deformation, mm; $h_{0}$ is the initial height of the sample, mm.

To calculate the axial stress, the average cross-section area of sample $A_{a}$ should be amended with the following equation, because the cross-section area is variable during loading:

(2) $$A_{a}=\frac{A_{0}}{1-0.01\varepsilon }$$

where $A_{a}$ represents the cross-section area during loading, mm^2^; $A_{0}$ is the initial cross-section area of the sample, mm^2^; ε is the axial strain.

The axial stress can be calculated based on the coefficient of the load cell gauge and the result of the load cell gauge as follows:

(3) $$\sigma =\frac{CR}{A_{a}}\times 10^{-3}$$

where σ is the axial stress, kPa; C represents the coefficient of load cell gauge, N/0.01 mm; R represents the result of load cell gauge, 0.01 mm.

## 3. Results and Discussions

### 3.1. Mechanical Behavior Under Combined Fiber-Cement Stabilization

**Figure 5:**
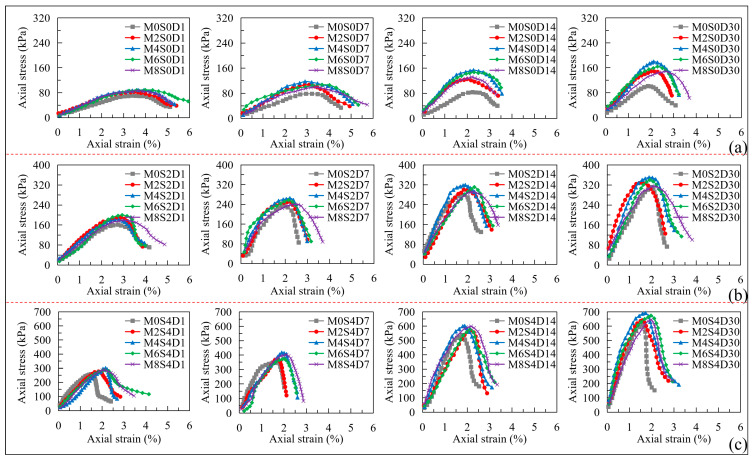
Variations of axial stress versus axial strain of treated sandy clayey silt under different lignin fiber contents and curing periods: (**a**) cement content of 0%; (**b**) cement content of 2%; (**c**) cement content of 4%.

Lignin fiber is a crucial organic fiber stabilizer and has a great influence on soil properties. To evaluate the influence of lignin fiber on the improvement of sandy clayey silt, the UCS tests are conducted with different contents (0%, 2%, 4%, 6%, and 8%) of lignin fiber in mixed sandy clayey silt. The variations of axial stress versus axial strain of treated sandy clayey silt under different lignin fiber contents and curing periods are shown in Figure 5.

The stress-strain curves of treated sandy clayey silt with different lignin fiber contents are significantly different. As shown in Figure 5a, when the cement content is zero, the peak strength of treated sandy clayey silt mixed is not significant with the increase of the curing period. When the curing time is more than 14 days, the axial stress peak of M4 is the highest. The peak strength of treated sandy clayey silt mixed with different contents of lignin fiber is approximately the same when the samples are initially assembled (1-d cure). During curing, the mechanical property of treated sandy clayey silt gradually transformed from strain-softening to strain-hardening as the axial strain decreased when the samples reached the critical state. The peak strength increases with the increase of lignin fiber content (M ≤ 4%) when the curing period is up to 7 days. For cement-free samples (*S* = 0%), the axial strain increases with the increase in lignin fiber content due to enhanced fiber-soil interlocking and stress redistribution. Curing periods (1–30 days) exhibit negligible influence on strain behavior in the absence of cement, as no hydration-driven stiffening occurs. The same phenomena are found with the cement contents of 2% and 4%, as shown in Figure 5b and Figure 5c, respectively. The reasons for this may be as follows: (1) the structure of treated sandy clayey silt benefits from the relatively high tensile strength and ductility of lignin fiber networks, which counteract the material’s intrinsic low compressive resistance by redistributing stresses and delaying crack formation [13,14]; (2) lignin fiber will sufficiently contact with the circumjacent soil particles during the curing time, thereby increasing the bond between soil particles and making them more stable; (3) the large contact area between the lignin fiber and soil causes an increase in the internal shear resistance and improves crack resistance inside the soil, which leads to the transformation of treated sandy clayey silt from brittle towards ductile [34].

**Figure 6:**
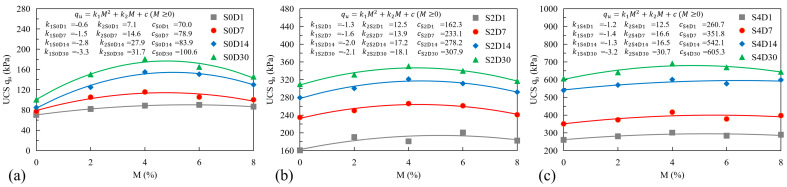
Influence of different factors on UCS of treated sandy clayey silt (**a**) Cement content 0%; (**b**) Cement content 2%; (**c**) Cement content 4%.

It should be noted that with the increase of the lignin fiber content, the peak strength of treated sandy clayey silt with different cement contents first increases then decreases. The peak strength initially rises with lignin fiber content (≤4%) due to stress redistribution and enhanced friction. Beyond 4%, fiber aggregation increases void ratios and disrupts cement hydration, shifting failure mechanisms to frictional slippage and reducing strength. The curves of the peak strength of treated sandy clayey silt under different lignin fiber and cement contents are shown in Figure 6. Compared with the samples with a lignin fiber content of 0%, the compressive strength of treated sandy clayey silt increased by 50%, 80%, 65%, and 45% when the lignin fiber content was 2%, 4%, 6%, and 8%, respectively. The peak strength of treated sandy clayey silt reaches the peak when the lignin fiber content is 4%. The quadratic terms between peak strength and lignin fiber content can be made as follows:

(4) $$q_{u}=k_{1}M^{2}+k_{2}M+c\;\;\;(M\geq 0)$$

where $k_{1}$ is the slope of the fitted curve that represents the ratio of compressive strength increment to the lignin fiber increment; M is the content of lignin fiber. *k*_1_ governs the curvature of the parabola, representing the diminishing returns or adverse effects of excessive fiber content. *k*_2_ is a linear term reflecting the initial positive contribution of fiber to strength. *C* is an intercept term representing the baseline strength of untreated soil. The values of $k_{1}$, $k_{2}$, and *c* under different lignin fiber contents, cement contents, and curing periods are shown in Figure 6.

As mentioned above, while the addition of lignin fiber enhances interparticle bonding, exceeding the critical threshold of 4% (identified in this study) shifts the dominant mechanism of compressive strength from cohesive cementation (governed by electrochemical bonds) to toughness and internal frictional resistance (driven by fiber-soil interlocking and particle rearrangement) [13]. The cohesive property is attributed to the elastic stiffness modulus, which could contribute to compressive strength. The ductility of treated sandy clayey silt is built by the capability of lignin fiber with higher interfacial friction around the soil-fiber matrix [34]. Moreover, the more lignin fiber content is mixed, the greater the void ratio will be, which will reduce the compressive resistance of the sample. To reveal the interfacial structure between lignin fiber and soil particles, SEM-EDX tests are conducted.

**Figure 7:**
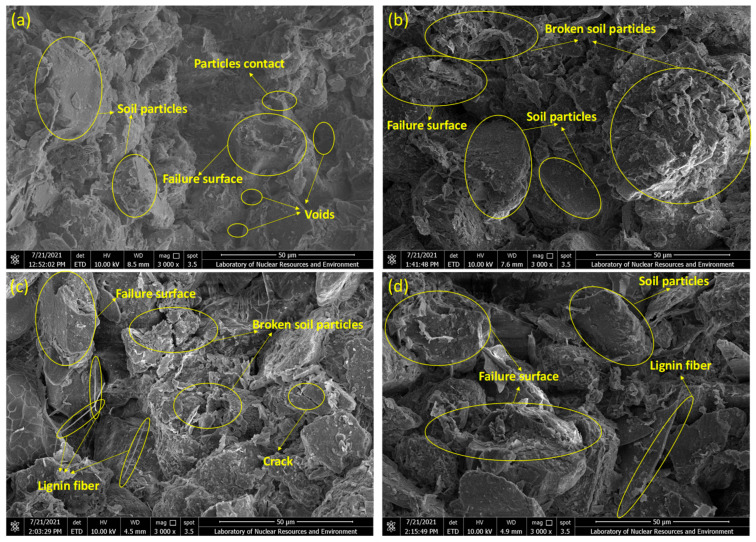
SEM-EDX micrographs of (**a**) M0S0 (no lignin fiber M and cement S); (**b**) M4S0 (4% lignin fiber M and no cement S); (**c**) M6S0 (6% lignin fiber M and no cement S); and (**d**) M8S0 (8% lignin fiber M and no cement S) under 3000 magnifications.

Figure 7 presents the changes in microstructures of the treated sandy clayey silt samples, M0S0 to M8S0. The failure surface image at 50 μm resolution was obtained from Scanning Electron Microscopy equipped with an energy-dispersive X-ray analyzer (EDX) (SEM-EDX) at 3000 magnifications. Figure 7a represents the microstructures of the original sandy clayey silt M0S0, which means the contents of the lignin fiber and cement are zero. It can be compared with other microstructures of the treated sandy clayey silt samples. As shown in Figure 7b,c, a series of unbroken soil particles contact each other with large amounts of voids across the failure surface. The connection of particles almost relies on the silt and clay particles, which reflects the point contact and surface contact. Lignin fiber as the linear structural material could improve the contact area between soil particles, and the ductility of samples during compression is enhanced [13,16,40]. Lignin fiber across the gaps between soil particles represents the failure surface (Figure 7d) on which the void area would decrease, which would also improve the compressive strength. Moreover, the broken soil particles occupy a certain percentage at the failure surface, leading to the improvement of soil ductility. Therefore, based on the SEM-EDX micrographs, the angular distribution of void orientation frequency, void area ratio, and average diameter of the void are discussed.

### 3.2. Role of Curing Time on Strength-Ductility Transition

**Figure 8:**
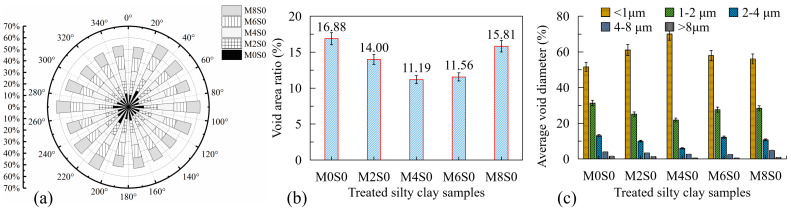
(**a**) Angular distribution of void orientation frequency; (**b**) the ratio of void area to total area at failure surface; (**c**) average void diameter percentage at failure surface, under different contents of lignin fiber without cement.

As an orientation index, void orientation frequency represents the distribution intensity of voids in a certain direction. To analyze the directive property of the void after the incorporation of lignin fibers in the sandy clayey silt, the void orientation frequency of treated sandy clayey silt after compressive failure is evaluated for different lignin fiber contents on the damaged surface, as shown in Figure 8a. The void orientation frequency in sandy clayey silt without lignin fiber is nonuniform, as it is concentrated on 20–40°, 80–100°, and 160–180°. Nevertheless, with the increase of lignin fiber content (when lignin fiber content is less than 6%), the void appears more uniform as the distribution intensity of the orientation frequency approximates a circle. When the lignin fiber content is up to 8%, the void orientation frequency mainly focuses on 30–110° and 210–290°. The tendency of void orientation frequency is similar to that of void area ratio (Figure 8b). The optimal lignin fiber content of 4% balances fiber reinforcement and matrix integrity. Beyond this threshold, fiber aggregation increases void ratios (Figure 8b), while limited water availability impedes cement hydration. These factors collectively reduce cohesive strength and shift failure mechanisms to frictional slippage, as evidenced by SEM-EDX and stress-strain analyses. The peak UCS and axial failure strain at 4% lignin fiber content (M4S0) correlate with the highest fine void percentage (<1 μm, Figure 8c) and homogeneous void orientation (Figure 8a). This microstructure enhances fiber-soil interlocking and stress redistribution, increasing ductility. Conversely, the decline in fine voids and rise in macrovoids (>8 μm) at 8% fiber content (M8S0) disrupt the fiber-soil matrix, reducing strength and ductility due to localized stress concentrations [13,15].

As mentioned above, the lignin fiber content in the improvement of sandy clayey silt has a limit value (*M* = 4%), and when M exceeds 4%, the void ratio of treated sandy clayey silt increases. As shown in Figure 8b, the minimum void area ratio at the failure surface is 11.19% of M4S0, and then increases to 15.81% of M8S0, giving the same tendency to UCS (Figure 6). The percentages of average void diameters at the failure surface of treated silty lacy under different lignin fiber contents are shown in Figure 8c. With the increase of lignin fiber content, the percentage of average void diameter, which is less than 1 μm, increases first, then decreases, and the maximum exists when lignin fiber content is 4%. However, the percentages of average void diameters of 1–2, 2–4, 4–8, and >8 μm show the contrary tendency (e.g., decreasing first, then increasing). The reasons may be as follows: (1) lignin fiber could enhance the connection between soil particles and improve the ductility of samples, which leads to the increase of broken soil particles and the fine void increasing [45]; (2) lignin fiber relatively improves the density of sandy clayey silt; however, the treated sandy clayey silt becomes loose when the lignin fiber content exceeds 4% [46]; (3) based on the principle of macro void preferential change, the macro voids are significantly reduced and the change of fine voids is minor, meaning that the void distribution is gradually homogenized [47,48].

Cement, as an improved material, is widely utilized in stabilizing soil. After the cement inclusion, cementing and curing actions between cement and soil particles will enhance the resistance to the applied load. To evaluate the effect of cement on the enhancement of strength and failure strain of treated sandy clayey silt, the cement contents of 0%, 2%, and 4% are mixed into the sandy clayey silt and lignin fiber-stabilized sandy clayey silt in the present study.

**Figure 9:**
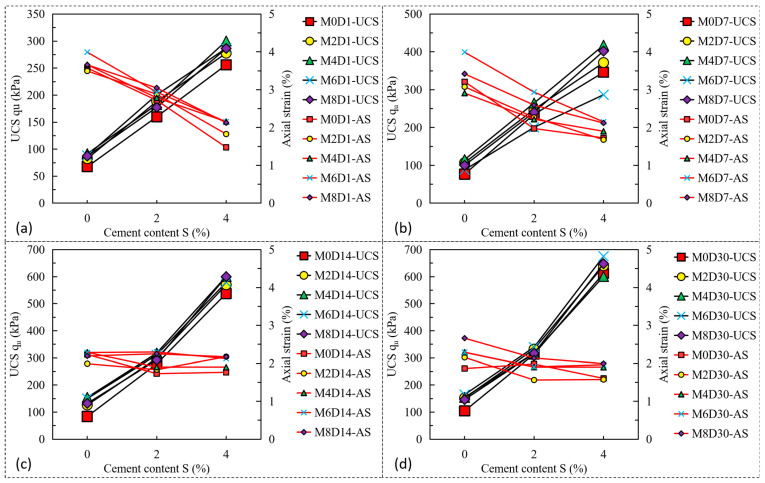
Effect of cement content on UCS and axial failure strain (AS) of treated sandy clayey silt under different curing periods: (**a**) 1-d; (**b**) 7-d; (**c**) 14-d; (**d**) 30-d.

Figure 9 plots the relationships between cement content, UCS, and axial failure strain (AS) under different curing periods. The results show that with the increase of cement content, the UCS of treated sandy clayey silt increases, and the AS decreases with the linear relationships. The increase in cement from 2% to 4% leads to an increase in UCS from 159.90 kPa (M0S2D1) to 256.02 kPa (M0S4D1). A comparison has been made between the samples under different curing periods that shows that the UCS of fiber-cement-stabilized sandy clayey silt increases with the increasing the curing period; however, the axial failure strain performs the opposite tendency, with the stabilized materials reaching peak strength with lesser axial strain. For example, the increments of UCS of M0S0, M0S2, and M0S4 under curing periods of 7-d and 30-d are 13.94%, 46.84%, and 35.18% (7-d) and 54.90%, 96.20%, and 139.24%, respectively, compared to the 1-d cure sample. By contrast, with the exception M0S4, the AS decrements of M0S0 and M0S2 under curing periods of 7-d and 30-d are 10.19% and 27.44% (7-d) and 91.79% and 37.24% (30-d), respectively. The same phenomenon is found when the sandy clayey silt is treated with an increase in cement content. When the curing period is more than 14-d, the AS of treated sandy clayey silt is approximately the same as the increase in curing period, changing the behavior of the treated material from ductile to brittle [1,2]. The cement hydration dominates, forming a rigid matrix that restricts particle mobility and fiber bridging. Microcrack propagation in the brittle matrix overrides lignin fiber’s ductilizing effects, culminating in strain-softening behavior (Figure 9).

### 3.3. Synergistic Microstructural Mechanisms

**Figure 10:**
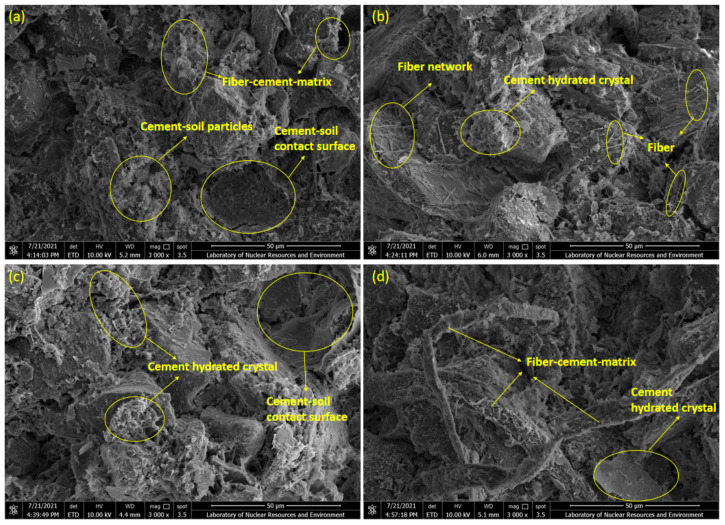
SEM-EDX images of lignin fiber-cement treated sandy clayey silt: (**a**) M2S4D30 (2% lignin fiber M and 4% cement S, 30-day curing); (**b**) M4S4D30 (4% lignin fiber M and 4% cement S, 30-day curing); (**c**) M6S4D30 (6% lignin fiber M and 4% cement S, 30-day curing); (**d**) M8S4D30 (8% lignin fiber M and 4% cement S, 30-day curing) under 3000 magnifications.

The enhancement of strength of treated sandy clayey silt with cement inclusion could be explained by the fact that the bond strength of cement is usually larger than that of sandy clayey silt, as the hydration reaction of cement leads to the cement-hydrated crystal surrounding soil particles, thereby improving the cohesive strength of the soil. When sandy clayey silt is treated with both cement and lignin fiber, more stable characteristics of treated sandy clayey silt will be obtained through the interlocking force and interfacial interaction between lignin fibers, cement hydrated crystal, and soil particles. Figure 10 shows the SEM-EDX images of the failure surface of treated sandy clayey silt of M2S4D30, M4S4D30, M6S4D30, and M8S4D30. It can be seen that when adding both lignin fiber and cement to soil, cement-soil particles attach to the surface of the lignin fibers, which allows the fiber-cement matrix to improve the interlocking force. Moreover, due to the random distribution, the fiber networks are created by attaching to the soil surface. Soil particles are also surrounded by the cement-hydrated crystal, which leads to the detachment of soil particles across the cement-soil contact surface and less occurrence of broken particles. However, the effect of cement on the UCS of sandy clayey silt treated by both lignin fiber and cement during the long curing period is insignificant. It could be explained that the brittle behavior influenced by cement inclusion plays the main role in compressive failure instead of ductile behavior with a low tensile strength of lignin fiber. The brittleness of cement-stabilized sandy clayey silt arises from a rigid hydration network (Figure 10b), consistent with findings in cement-treated clays [41]. However, lignin fiber introduces a unique duality: at ≤4% cement, fibers delay crack propagation via bridging, whereas higher cement contents (>4%) prioritize brittle matrix formation, overriding fiber efficacy. This contrasts with polypropylene-fiber systems [12], where ductility persists at elevated binder levels, highlighting lignin’s limitations in highly cemented matrices. While lignin fiber content increases in Figure 10c,d (6–8%), individual fibers are less visible due to clustering and encapsulation by cement hydration products.

**Figure 11:**
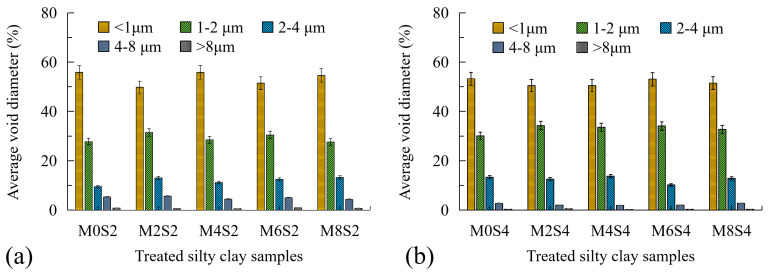
Average void diameter percentage of treated sandy clayey silt with cement inclusion of (**a**) 2%; (**b**) 4% at failure surface after a 30-d cure.

Figure 11 depicts the percentage of different average void diameters of treated sandy clayey silt at the failure surface after a 30-d cure. With the comparison results of Figure 8c, the ratios of soil particles with diameters of less than 1 μm and 1–2 μm of treated sandy clayey silt due to the inclusion of cement are approximately the same, and the effect of lignin fiber on broken soil particles could be neglected. It could be explained by the fact that when adding cement to fiber-treated sandy clayey silt, the cement-hydrated crystal will prevent the soil particles from crushing what is around it after a long curing period. Moreover, the compressive characteristic of strain hardening of cemented soil leads to a smoother failure surface, as its behavior changes from ductile to brittle. Cement hydration forms a rigid skeleton (Figure 10) that elevates UCS but reduces ductility (Figure 9). The brittle failure mode is attributed to the dominance of cementitious bonds over fiber-mediated ductility, as evidenced by the suppressed particle crushing (Figure 11) and strain-softening behavior (Figure 5c) [6,8].

## 4. Conclusions

This study investigated the UCS and microstructural performance of sandy clayey silt treated with lignin fiber and cement. The influences of several factors, including lignin fiber content, cement content, and curing time, were examined. Moreover, the microstructure and interaction of the lignin fiber-cement-soil particles in treated sandy clayey silt, such as angular distribution of void orientation frequency, the ratio of void area to the total area, and average void diameter percentage at failure surface, were analyzed using Electron Microscopy equipped with an energy-dispersive X-ray analyzer (EDX) (SEM-EDX). Based on the experimental results, the following main conclusions can be drawn:

(1) The mechanical properties of treated sandy clayey silt transitioned from strain-softening to strain-hardening during curing, highlighting the synergistic role of lignin fiber (enhancing ductility) and cement (improving stiffness) in stabilizing waste soils. This dual stabilization approach aligns with sustainable construction goals by repurposing engineering waste soils into viable backfill materials, reducing reliance on virgin aggregates and lowering carbon-intensive cement usage.
(2) Lignin fiber content exhibited a threshold value of 4% for optimal strength enhancement. Beyond this limit, increased void ratios (up to 15.8%) weakened the soil matrix, underscoring the need for balanced fiber incorporation to maximize resource efficiency. This finding supports sustainable practices by demonstrating how low-cost, biodegradable fibers can partially replace cement (up to 4% in this study) while maintaining performance, thereby lowering material costs and embodied carbon.
(3) Cement content linearly improved UCS (by 96–139% over 30 days) but reduced axial failure strain (by 37–91%), emphasizing the trade-off between strength and brittleness. For sustainable infrastructure, this suggests that cement dosage should be minimized where ductility is critical (e.g., seismic zones), with lignin fiber compensating for strength loss, a strategy that extends the service life of waste soil applications while mitigating brittle failure risks.
(4) Microstructural analysis revealed that lignin fiber-cement matrices formed interlocking networks, reducing particle detachment and homogenizing void distributions (e.g., <1 μm voids increased by 22% at 4% fiber). This micro-to-macro behavior validates the viability of lignin-fiber stabilization as a scalable, eco-friendly technique for repurposing silty waste soils in subgrades or embankments.
(5) While this study demonstrates the short-term efficacy of lignin fiber-cement stabilization, long-term durability under field conditions (e.g., cyclic loading, freeze-thaw, and chemical leaching) remains unverified. Future research should investigate (i) the biodegradation kinetics of lignin fibers in soil-cement systems, (ii) lifecycle carbon footprint analysis comparing this method to conventional stabilization, and (iii) field trials to assess scalability in diverse geoenvironments. Additionally, exploring alternative biofibers (e.g., rice husk, cellulose) could further advance sustainable waste soil reuse.

## Figures and Tables

**Table 1:** Geotechnical index properties of sandy clayey silt.

Density (g/cm^3^)	Moisture Content (%)	Gravity	Liquid Limit (%)	Plastic Limit (%)	Plasticity Index
1.66	24.02	2.69	34.00	20.10	13.9

**Table 2:** Particle size distribution of sandy clayey silt.

Particle Radius/mm			Characteristic Grain/(μm)				C_u_	C_c_
0.075–2	0.005–0.075	<0.005	d_10_	d_30_	d_60_	d_90_		
6.4%	83.97%	9.63%	7.472	16.01	39.75	65.75	7.8	1.31

**Table 3:** Test scheme of permeability coefficient.

Moisture Content (%)	Lignin Fiber Content M(%)	Cement Content S(%)	Curing Time (D)
W24	M0	S0	D30
		S2	
		S4	
	M2	S0	
		S2	
		S4	
	M4	S0	
		S2	
		S4	
	M6	S0	
		S2	
		S4	
	M8	S0	

## Data Availability

The raw data supporting the conclusions of this article will be made available by the authors without undue reservation.
